# Therapy management and outcome of acute hydrocephalus secondary to intraventricular hemorrhage in adults

**DOI:** 10.1186/s41016-024-00369-0

**Published:** 2024-06-03

**Authors:** Chaoyang Wang, Jianuo Bai, Qiheng He, Yuming Jiao, Wenqian Zhang, Ran Huo, Jie Wang, Hongyuan Xu, Shaozhi Zhao, Zhiyou Wu, Yingfan Sun, Qifeng Yu, Jinyi Tang, Xianwei Zeng, Wuyang Yang, Yong Cao

**Affiliations:** 1 Department of Neurosurgery, Shenzhen Qianhai Shekou Free Trade Zone Hospital, Shenzhen, China; 2 Department of Neurosurgery, The Johns Hopkins Hospital, 1800 Orleans Street Suite 6007, Baltimore, MD 21287 USA; 3 Department of Neurosurgery, Beijing Tiantan Hospital, Capital Medical University, No.119 South West 4th Ring Road, Beijing, China; 4 Department of Neurosurgery, National Research Center for Rehabilitation Technical Aids, Beijing, China; 5 Department of Neurosurgery, Rehabilitation Hospital, National Research Center for Rehabilitation Technical Aids, Beijing, China; 6 Key Laboratory of Neuro-Functional Information and Rehabilitation Engineering of the Ministry of Civil Affairs, Beijing, China

**Keywords:** Hydrocephalus, Intracerebral hemorrhage, Intraventricular hemorrhage, Management, Systematic review

## Abstract

**Background:**

Intraventricular hemorrhage (IVH) refers to bleeding within the brain’s ventricular system, and hydrocephalus is a life-threatening complication of IVH characterized by increased cerebrospinal fluid accumulation in the ventricles resulting in elevated intracranial pressure. IVH poses significant challenges for healthcare providers due to the complexity of the underlying pathophysiology and lack of standardized treatment guidelines. Herein, we performed a systematic review of the treatment strategies for hydrocephalus secondary to IVH.

**Methods:**

This systematic review was prospectively registered with PROSPERO (CRD42023450786). The search was conducted in PubMed, Cochrane Library, and Web of Science on July 15, 2023. We included original studies containing valid information on therapy management and outcome of hydrocephalus secondary to primary, spontaneous, and subarachnoid or intracranial hemorrhage following IVH in adults that were published between 2000 and 2023. Glasgow Outcome Scale (GOS) or modified Ranking Scale (mRS) scores during follow-up were extracted as primary outcomes. The risk of bias was assessed using the Newcastle–Ottawa Scale for Cohort Studies or Cochrane Risk of Bias 2.0 Tool.

**Results:**

Two hundred and seven patients from nine published papers, including two randomized controlled trials, were included in the analysis. The GOS was used in five studies, while the mRS was used in four. Seven interventions were applied, including craniotomy for removal of hematoma, endoscopic removal of hematoma with/without endoscopic third ventriculostomy (ETV), traditional external ventricular drainage (EVD), and various combinations of EVD, lumbar drainage (LD), and intraventricular fibrinolysis (IVF). Endoscopic removal of hematoma was performed in five of nine studies. Traditional EVD had no obvious benefit compared with new management strategies. Three different combinations of EVD, LD, and IVF demonstrated satisfactory outcomes, although more studies are required to confirm their reliability. Removal of hematoma through craniotomy generated reliable result. Generally, endoscopic removal of hematoma with ETV, removal of hematoma through craniotomy, EVD with IVF, and EVD with early continuous LD were useful.

**Conclusion:**

EVD is still crucial for the management of IVH and hydrocephalus. Despite a more reliable result from the removal of hematoma through craniotomy, a trend toward endoscopic approach was observed due to a less invasive profile.

**Supplementary Information:**

The online version contains supplementary material available at 10.1186/s41016-024-00369-0.

## Background

Intraventricular hemorrhage (IVH) refers to bleeding within the ventricular system of the brain [1]. IVH is mostly secondary to intracerebral hemorrhage (ICH) and subarachnoid hemorrhage (SAH) in adults as opposed to a primary condition [2]. Approximately 40% of patients with ICH develop IVH, and 51–89% of patients with IVH are complicated with hydrocephalus [3–5], which is one of the most serious complications associated with IVH due to intracranial pressure elevation from cerebrospinal fluid (CSF) accumulation in the ventricles. The management of hydrocephalus secondary to IVH in adults poses significant challenges for healthcare providers due to the complexity of the underlying pathophysiology and lack of standardized treatment guidelines. IVH and secondary hydrocephalus are both independent risk factors for poor outcomes among adults with ICH [6].

Understanding the pathophysiology of hydrocephalus secondary to IVH is crucial for effective management. IVH results in the accumulation of blood within the ventricles, disrupting the normal flow and absorption of CSF. The presence of blood products triggers inflammatory response, leading to a fibrotic reaction in the ventricles and obstructing the flow of CSF through the narrow passages [7]. This obstruction can significantly increase intracranial pressure, potentially leading to neurological complications and permanent brain damage if not promptly managed. A review conducted in 2017 recommended the management of IVH-associated hydrocephalus with external ventricular drainage (EVD) [8]. However, treatments and outcomes of IVH-associated hydrocephalus among adults have been understudied, and appropriate management strategies may lead to better outcomes. A systematic review was performed previously to address this issue but has focused on outcomes among neonates only [9]. Therefore, we performed this systematic review to summarize and analyze data concerning the treatment and outcome of IVH-associated hydrocephalus in adults. We aimed to compare different treatment strategies and their outcomes, providing an overview of different management strategies available for this challenging condition.

## Methods

This systematic review was prospectively registered with PROSPERO (CRD42023450786). We followed the 2020 Preferred Reporting Items for Systematic Reviews and Meta-Analysis checklist to conduct our review [10].

## Search strategy and selection criteria

The search strategy was developed after discussion among the authors. Search terms included a combination of Medical Subject Headings and text words indicative of (1) hydrocephalus and (2) IVH; the full search strategy is listed in Additional file 1: Appendix S1. The final search was conducted in PubMed, Cochrane Library, and Web of Science on July 15, 2023. We included original studies containing valid information on therapy management and outcome of hydrocephalus secondary to primary or spontaneous SAH and ICH following IVH in adults that were published between 2000 and 2023. Studies were excluded if they were reviews, technique notes, case reports, case series, editorials, studies not in English, studies on animals, studies without valid full texts, studies in which we were unable to separate patients with different etiologies, studies in which we were unable to extract adult patients, and studies without valid information on outcomes.

## Data extraction and quality assessment

For all nine included studies, variables including the author, year, type of study, region, number of patients, intervention, follow-up, measurement, outcome score, and mortality were extracted. Outcomes were defined as different descriptions according to those used in the original studies. In studies of patients with multiple etiologies, patients were separated by etiology. We only extracted data of patients with primary IVH, spontaneous IVH, hypertensive IVH, and IVH secondary to ICH/SAH. Due to study heterogeneity, the Cochrane Risk of Bias 2.0 Tool was used to assess the quality of randomized controlled studies, and the Newcastle–Ottawa Scale for Cohort Studies (NOS) was used to assess the quality of cohort studies included [11, 12].

## Results

### Study selection and characteristics

**Fig. 1:**
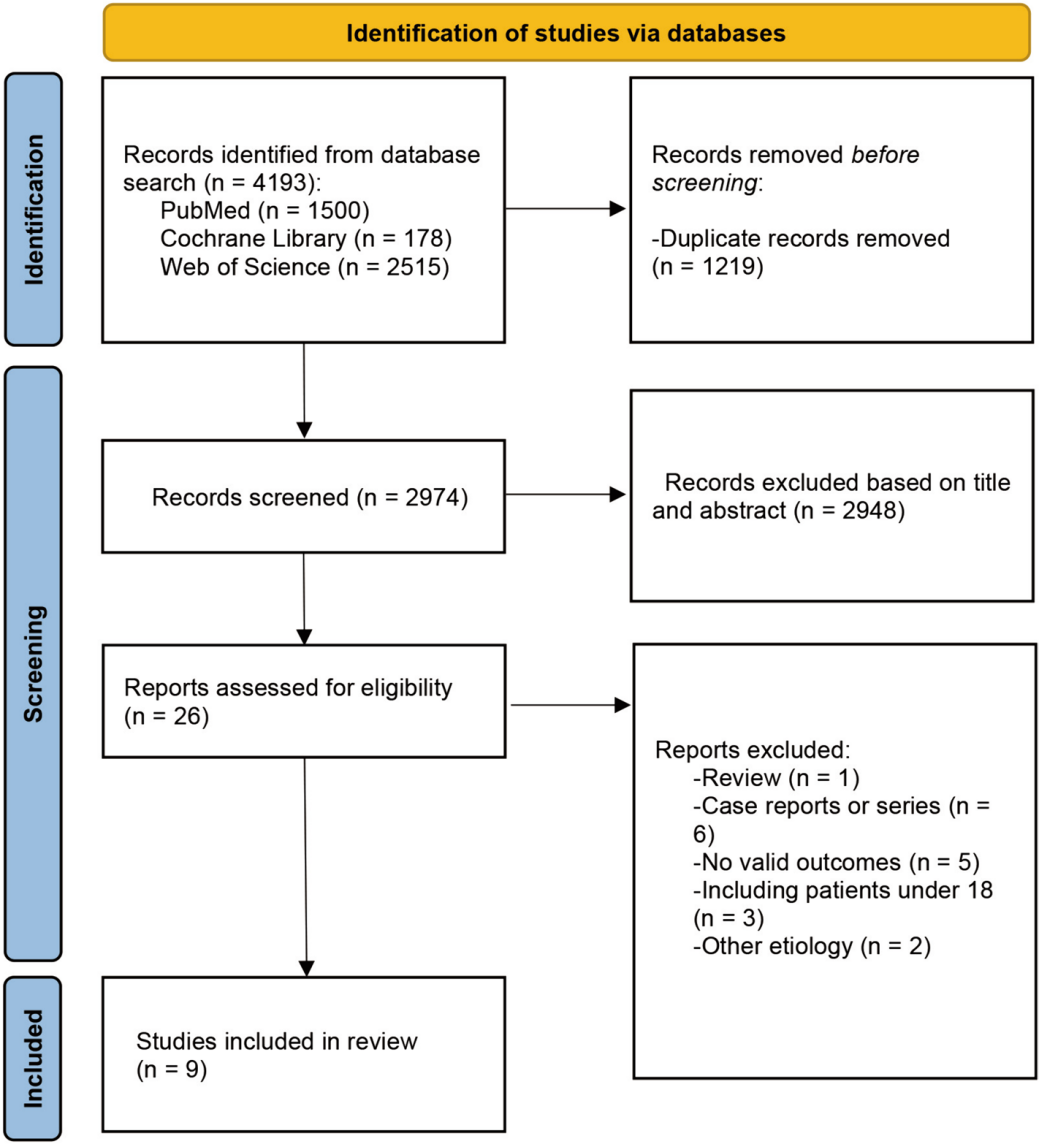
Flow-gram of the study

There were 2974 studies identified after literature search and removing duplications. There were 26 publications left for full-text review from title and abstract screening performed independently by two reviewers and discussion. Full-text review resulted in the exclusion of 17 studies, which included 1 review, 6 case reports or series, 5 reports with no valid outcomes, 3 reports from which we were unable to extract adult patients, and 2 reports of patients with other etiologies (Additional file 1: Appendix S2). Finally, nine articles met the inclusion criteria. The findings of these studies are summarized in Table 1. The flow chart of the study selection process is shown in Fig. 1.

**Table 1:** Study characteristics and findings

Study, year	Country	Study type	Population	Number of patients	Age, year	Interventions	Follow-up, month	Measurement	Outcome, score	Mortality, %	Conclusion
Wang et al., 2006 [13]	China	Prospective	Single-center	18	63.5	Physical removal of hematoma through craniotomy, septostomy approach, and EVD	6	GOS	3.83	5.6	Physical removal of the clot through an open exposure of the lateral ventricle approaching it from above through the corpus callosum made a rather impressive success rate
Yadav et al., 2007 [14]	UK	Prospective	Multi-center	25	-	Endoscopic removal of hematoma and ETV for hydrocephalus	6	GOS	3.32	24	Encouraging results had been shown thorough endoscopic management on hypertensive IVH patients with obstructive hydrocephalus
Hamada et al., 2008 [15]	Japan	Retrospective	Single-center	15	62	Endoscopic removal of hematoma and EVD	-	mRS	3.47	0	Endoscopy is of importance in the management of non-communicative hydrocephalus
Staykov et al., 2009 [16]	Germany	Prospective	Single-center	32	61	Treatment algorithm of IVF, EVD, and early application of LD	6	mRS	62.5% with good outcome (0–3)	15.6	First prospective study investigating combined treatment of IVF, EVD, and LD
Chen et al., 2011 [17]	China	Randomized controlled trial	Single-center	48	63.9	Endoscopic removal of hematoma and following EVD /traditional EVD	3	GOS	3.08 vs 3.33	20.8 vs 16.6	Endoscopic surgery had a significant lower incidence of shunt-dependent hydrocephalus and a shorter ICU stay compared with EVD surgery, and this can decrease the need for permanent VP shunt in IVH caused by thalamic hemorrhage
Wang et al., 2013 [13]	China	Randomized controlled trail	Multi-center	45	55.4	EVD and IVF	1	GOS	3.36	15.6	EVD plus EVT from the ipsilateral ventricle had faster blood clearance in the third and fourth ventricles than in the contralateral ventricle. Clinical outcome were similar in two strategies. Also, this is the first study investigating the effect of catheter location of EVD on patients’ outcome
Xia et al., 2014 [18]	China	Prospective	Single-center	8	68	EVD and early CLD	6	GOS	3.62	13	First prospective study providing insight into the safety, feasibility, and potential benefit of combining early CLD with EVD in moderate to severe IVH regardless of the presence of obstructive hydrocephalus
Obaid et al., 2015 [19]	Cananda	Retrospective	Single-center	8	58	Endoscopic removal of hematoma and ETV for hydrocephalus	21.3	mRS	3.5	37.5	ETV with or without endoscopic clot evacuation is a safe and effective method to treat IVH-related obstructive hydrocephalus
Ogiwara et al., 2021 [20]	Japan	Retrospective	Multi-center	8	54.4	Endoscopic removal of hematoma and ETV for hydrocephalus	At discharge	mRS	3	0	Tailor-made endoscopic strategy revealed a good hematoma evacuation rate and potential for improved functional outcome

*EVD* external ventricular hemorrhage, *GOS* Glasgow Outcome Scale, *ETV* endoscopic third ventriculostomy, *mRS* modified Rankin Scale, *IVF* intraventricular fibrinolysis, *LD* lumber drainage, *CLD* continuous lumber drainage

All included studies were interventional. Of these nine studies, four took place in China [13, 17, 18, 21], two in Japan [15, 20], one in Canada [19], one in Germany [16], and one each in India and the UK [14]. Four studies were prospective cohort studies [13, 14, 16, 18], three were retrospective cohort studies [15, 19, 20], and two were randomized controlled studies [17, 21]. Six studies were performed at a single center [13, 15–18, 22], while three were multi-center studies [14, 20, 21]. The mean age of the patients across studies was between 54 and 68 years. The sample size varied from 8 [18–20] to 48 patients [17]. In total, 207 patients were included in the review. The modified Rankin Scale (mRS) and Glasgow Outcome Scale (GOS) were the two outcome measurements used in all nine studies: the GOS was used in five studies [13, 14, 17, 18, 21], while the mRS was used in four [15, 16, 19, 20].

**Fig. 2:**
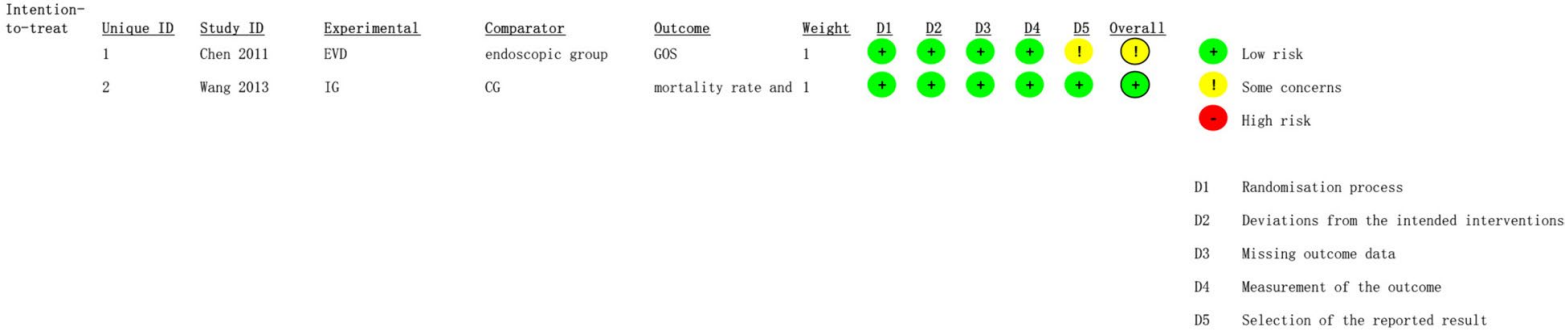
Overall quality assessment of included studies

The overall quality assessment results are shown in Table 2 and Fig. 2. One randomized controlled study [17] was rated as having some concerns in the domain of selection of the reported result, while the other randomized controlled study [21] was deemed low risk. Five [13, 14, 16, 18, 19] cohort studies were of good quality, one [20] was of fair quality, and one [15] was of poor quality due to the outcome domain.

**Table 2:** Risk of bias and quality assessment for cohort studies with the Newcastle–Ottawa Scale (NOS) for cohort studies

Source	Selection (scale, 1–4)	Comparability (scale, 1–2)	Outcome (scale, 1–3)	Total (1–9)	Study quality
Wang et al., 2006 [13]	3	1	3	7	Good
Yadav et al., 2007 [14]	3	1	3	7	Good
Hamada et al., 2008 [15]	3	1	1	5	Poor
Staykov et al., 2009 [16]	3	2	3	8	Good
Xia et al., 2014 [18]	3	1	3	7	Good
Obaid et al., 2015 [19]	3	2	3	8	Good
Ogiwara et al., 2021 [20]	3	1	2	6	Fair

### Management strategies and outcomes for IVH with hydrocephalus

Postoperative EVD was performed in all included studies. Several managements were performed in the included studies. All nine studies included various modalities of interventions, while two had attempted conservative management strategies [16, 21]. Wang et al. used direct physical removal of intraventricular blood clots through craniotomy, an interhemispheric trans-corpus callosal approach with septostomy, and EVD for hydrocephalus in 18 patients, with a mean GOS score of 3.83 after 6 months and mortality rate of 5.6% [13]. Endoscopic removal of intraventricular hematoma was performed in five studies with different outcomes [14, 15, 17, 19, 20]. Endoscopic removal of hematoma and endoscopic third ventriculostomy (ETV) for hydrocephalus were performed in 3 studies (41 patients) [14, 19, 20]. In the study by Yadav et al., the mean GOS score after 6 months was 3.32 [14]. In the study conducted by Obaid et al., the mean mRS score was 3.5 after a mean of 21.3 months [19]. The mean mRS score in the study conducted by Ogiwara et al. was 3.0 at discharge [20]. In the other two studies, endoscopic removal of hematoma was performed without ETV in 39 patients [15, 17]. In the study by Hamada et al., the mean mRS score was 3.47 with no mortality [15]. In the study by Chen et al., the endoscopic group achieved an average GOS score of 3.08; the mortality rate was 12.5% after 30 days and 20% after 90 days [17]. The overall mortality in the endoscopic-only group was 12.8%, which was lower than that of patients with endoscopic removal of hematoma and ETV. EVD without additional surgical intervention by Chen et al. in comparison with endoscopic removal of hematoma, with a GOS score of 3.33 after 90 days and mortality rate of 16.6% after 90 days in the EVD group and no significant difference was found [17]. Staykov et al. compared different management strategy including EVD, intraventricular fibrinolysis (IVF), and lumbar drainage (LD) in 32 patients, in which 56.3% had a good outcome (defined as an mRS score of 0–3) at 90 days and 62.5% had a good outcome at 180 days [16]. A combination of EVD and IVF was performed without LD by Wang et al. in 45 patients, where the average GOS score was 3.36 and the mortality rate was 15.6% at 30 days [21]. A combination of EVD and early continuous LD (CLD) was performed by Xia et al. in 8 patients, with average GOS scores after 3 and 6 months of 3.62 each and a mortality rate of 12.5% [18]. The results are summarized in Table 1.

### Comparison of outcomes across management strategies for IVH with hydrocephalus

**Fig. 3:**
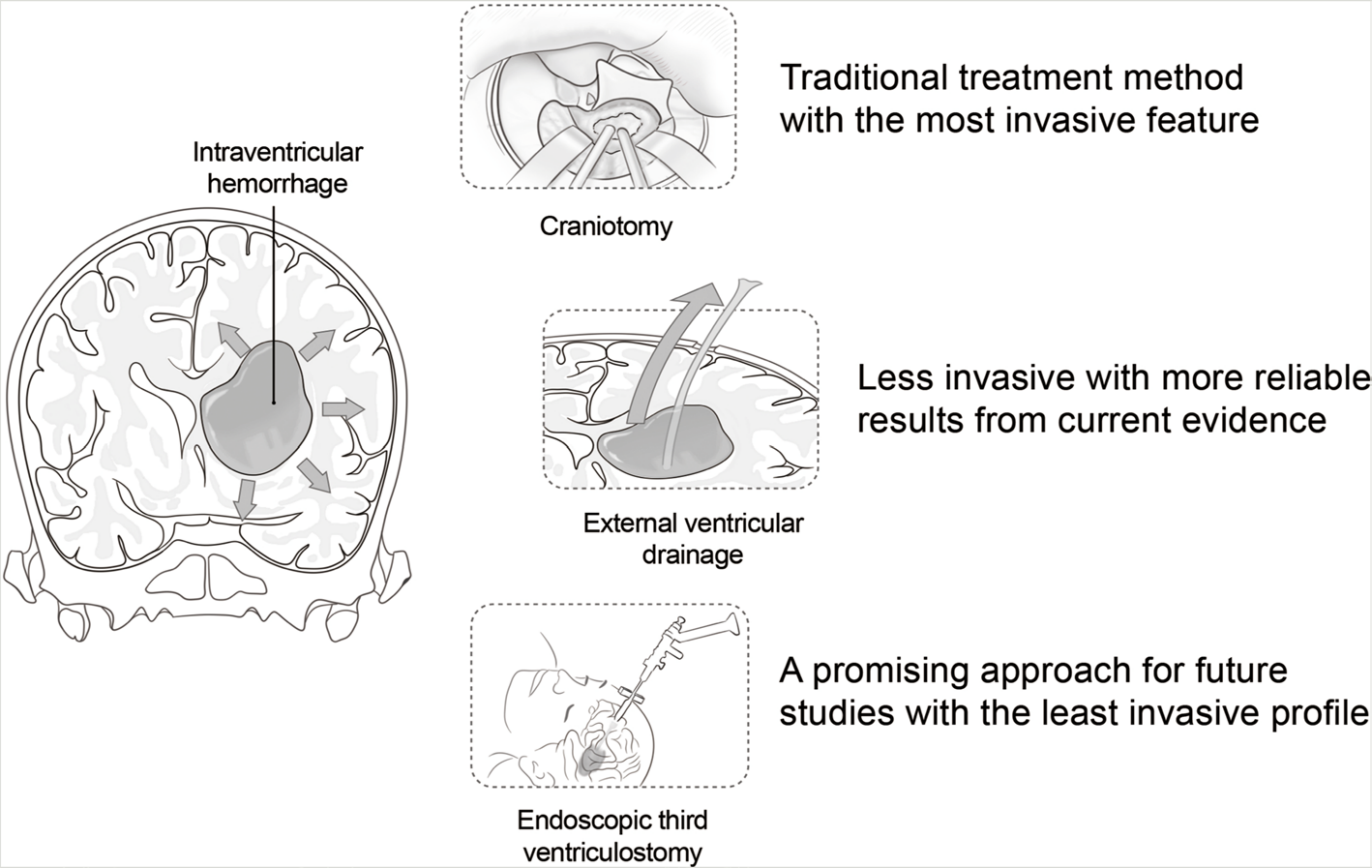
Comparison of different treatment modalities for intraventricular hemorrhage

Wang et al. reported a GOS score of 3.83 and a relatively low mortality of 5.6% in patients receiving physical removal through craniotomy [13]. Recently, a study by Chen et al. reported the average GOS score of 3.08 in patients receiving endoscopic removal of hematoma, which was comparable as that in the physical removal group [17]. In both studies by Obaid et al. and Ogiwara et al., endoscopic removal of hematoma combined with ETV was reported, with mRS scores ranging from 3 to 3.5, while holding a comparatively high mortality rate of 30% (Fig. 3) [19, 20].

### EVD and endoscopic removal of hematoma with/without ETV

EVD as a single modality performed by Chen et al. resulted in a mean GOS score of 3.33 and mortality rate of 16.6% [17]. In their study, EVD was compared with endoscopic removal of hematoma without ETV, resulting in no significant differences between the GOS scores and mortalities. The overall mortality rate of EVD was lower than that of endoscopic removal of hematoma with ETV but higher than the rate of endoscopic removal of hematoma without ETV. However, different sample sizes and different evidence levels of studies should be noticed. Endoscopic removal of hematoma with ETV had a higher mortality rate than endoscopic removal of hematoma without ETV. Due to heterogeneity of outcome measurements, the outcomes between managements could not be easily compared.

### Different combinations of EVD, IVF, and LD

Among different combinations of EVD, LD, and IVF, EVD and early CLD had a lower mortality rate of 12.5%, while the other combinations had a mortality rate of 15.6%. The combination of EVD and early CLD had a higher GOS score than the combination of EVD and IVF [18, 21]. However, due to the much smaller sample size and poorer evidence level, it is difficult to statistically compare these combinations. The combination of EVD, IVF, and LD led to 62.5% of patients receiving a good outcome (mRS score of 0–3) [16].

## Discussion

This systematic review included nine articles on IVH treatments and outcomes with secondary hydrocephalus published between 2000 and 2021. Seven categories of interventions, including physical removal of hematoma, endoscopic removal of hematoma with/without ETV, traditional EVD, and different combinations of EVD, LD, and IVF, were applied in these studies. Endoscopic removal of hematoma was performed in five of nine studies [14, 15, 17, 19, 20]. Although different outcomes were demonstrated, this is still a promising direction. Traditional EVD was not obviously superior to the other management strategies. The three different combinations among EVD, LD, and IVF resulted in impressive outcomes, although all three studies recommended further studies to confirm the reliability of the result. Physical removal of hematoma through craniotomy showed reliability, although its invasiveness should be considered. Generally, endoscopic removal of hematoma with ETV, physical removal of hematoma through craniotomy, EVD with IVF, and EVD with early CLD could be useful.

The initial management of IVH and secondary hydrocephalus begins with close monitoring of the patient’s clinical status, including regular neurologic assessments and frequent imaging studies to assess the extent of bleeding and development of hydrocephalus [23]. Medical management options rely on conservative measures such as the use of osmotic agents (e.g., mannitol or hypertonic saline) to reduce intracranial pressure and administration of diuretics (e.g., acetazolamide or furosemide) to decrease CSF production [24–28]. These measures attempt to temporize intracranial pressure while awaiting further interventions.

As per the guideline from the American Stroke Association/American Heart Association published in 2022, intraventricular thrombolysis (IVT) with EVD is primarily recommended in patients with IVH and hydrocephalus to reduce mortality [29]. Surgical treatments were dominant in our results, although more alternative approaches were developed and adopted in recent years for treating ICH with hydrocephalus. A series of IVH trials named Clot Lysis Evaluating Accelerated Resolution of Intraventricular Hemorrhage involved intraventricular injection of recombinant tissue plasminogen activator (rt-PA) [30, 31]. Clinical trials demonstrated that IVT with rt-PA injection facilitated clot dissolution [32, 33]. However, some studies indicate that clot lysis with rt-PA injection alone has no effect on the prevention of hydrocephalus in IVH. The efficacy of rt-PA injection in preventing hydrocephalus and improving neurological outcomes is currently being evaluated in a Phase 3 trial [34]. Among the surgical management strategies, EVD is often the initial step to provide immediate CSF diversion and relieve acute intracranial hypertension. EVD has shown effectiveness in reducing intracranial pressure and helping stabilize patients while awaiting definitive treatment options.

Definitive treatment options for IVH-associated hydrocephalus include the placement of a ventriculoperitoneal (VP) shunt or the use of ETV. VP shunting involves the insertion of a catheter from the ventricles to the peritoneal cavity, enabling CSF diversion and reducing intracranial pressure [35]. Despite its widespread use, VP shunting carries a risk of complications such as shunt malfunction, infection, and over-drainage, which may require further surgical interventions.

Alternatively, ETV offers a minimally invasive no-implant approach that creates an artificial connection between the ventricles and subarachnoid space, bypassing the obstructed pathway. ETV has gained popularity in recent years due to its potential advantages of reducing the need for lifelong shunt dependency and associated risks. However, its success can be affected by multiple factors, including the size and location of the obstructed ventricle [36, 37].

A relationship between IVH and cardiac disorders was observed in two studies. Garrett et al. suggested that elevated cardiac troponin levels were predictive of mortality in surgically treated patients with ICH and should be considered in management decisions [38]. Another retrospective study conducted in 2006 also showed similar findings [39]. In 2021, Lin et al. discovered that elevated cardiac troponin I level after the ictus of a ruptured intracranial aneurysm can predict the occurrence of major adverse cardiac events and unfavorable outcomes within 2 years after asymptomatic SAH [40]. The relationship between craniocerebral disease and cardiac markers provides a potential therapeutic direction for further studies.

We also listed the limitations associated with the included studies in this review. The sample size used by Wang et al. was too small for statistical analysis in this study [13]. A controlled study design and larger sample size were needed to draw definitive conclusions in the study by Staykov et al. [16]. Performing ETV and IVF could have reduced the need for shunt surgery in the study by Chen et al. [17]. Relatively larger sample size could have strengthened the statistical power in the study by Wang et al. [21]. A control group was needed to draw conclusions regarding the effect of treatment on outcomes in the study by Obaid et al. [19]. A control group and larger sample size were needed in the study by Xia et al. [18]. Larger sample size and functional outcome evaluations were needed in the study by Ogiwara et al. [20].

This systematic review has its strengths and limitations. To the best of our knowledge, this is the first systematic review on the management of IVH and hydrocephalus in adult patients. Two quality assessment tools were used, thereby improving accuracy. This review was conducted using PubMed as the single source, which might limit the sensitivity of capturing all existing literature, but a thorough discussion before literature search reached the consensus that the number of publications in PubMed was adequate for a systematic review. Due to the heterogeneity among study outcomes and the number of studies included, we were unable to conduct a meta-analysis. Furthermore, only articles in English were included, which may further lower sensitivity of finding relevant literature. Finally, there were only two randomized controlled studies in this review, and studies with higher evidence levels are needed.

## Conclusion

Our review aimed to comprehensively target the subject of therapy management and outcomes of hydrocephalus secondary to primary IVH in adults. To our knowledge, this is the first review to address IVH-associated hydrocephalous management in the adult population. Different treatments for IVH with hydrocephalus and the associated outcomes were compared. According to our findings, EVD remains as the mainstay management of IVH and hydrocephalus. Craniotomy for evacuation of hematoma generated reliable result, but alternative approaches such as endoscopic evacuation are being increasingly adopted due to the less invasive nature. Different combinations of EVD, IVF, and LD demonstrated satisfactory outcomes, although more studies with higher level of evidence should be performed to ascertain the current findings.

### Supplementary Information

Additional file 1: Appendix S1. Full search strategy in PubMed, Cochrane Library and Web of Science. Appendix S2. Detailed reasons for the excluded studies.
