# Supplementary material for: Therapy management and outcome of acute hydrocephalus secondary to intraventricular hemorrhage in adults
Source: Chin Neurosurg J. 2024 Jun 3;10:17. doi: 10.1186/s41016-024-00369-0 (PMC11149196; doi:10.1186/s41016-024-00369-0)
Supplement: Supplementary file 1 — Additional file 1: Appendix S1. Full search strategy in PubMed, Cochrane Library and Web of Science. Appendix S2. Detailed reasons for the excluded studies. [file 41016_2024_369_MOESM1_ESM.docx]

Supplementary Material

# Supplementary Data

**Appendix S1.** Full search strategy in PubMed, Cochrane Library and Web of Science.

**Search strategy in PubMed:** ((((((((((((((((((((((((((((Hydrocephalus[MeSH Terms]) OR (Hydrocephaly[Text Word])) OR (Cerebral Ventriculomegaly[Text Word])) OR (Cerebral Ventriculomegalies[Text Word])) OR (Ventriculomegalies, Cerebral[Text Word])) OR (Ventriculomegaly, Cerebral[Text Word])) OR (Communicating Hydrocephalus[Text Word])) OR (Hydrocephalus, Communicating[Text Word])) OR (Congenital Hydrocephalus[Text Word])) OR (Hydrocephalus, Congenital[Text Word])) OR (Hydrocephalus Ex-Vacuo[Text Word])) OR (Hydrocephalus Ex Vacuo[Text Word])) OR (Hydrocephalus Ex-Vacuos[Text Word])) OR (Obstructive Hydrocephalus[Text Word])) OR (Hydrocephalus, Obstructive[Text Word])) OR (Post-Traumatic Hydrocephalus[Text Word])) OR (Hydrocephalus, Post-Traumatic[Text Word])) OR (Post Traumatic Hydrocephalus[Text Word])) OR (Aqueductal Stenosis[Text Word])) OR (Aqueductal Stenoses[Text Word])) OR (Stenoses, Aqueducta[Text Word])) OR (Stenosis, Aqueductal[Text Word])) OR (Fetal Cerebral Ventriculomegaly[Text Word])) OR (Cerebral Ventriculomegalies, Fetal[Text Word])) OR (Cerebral Ventriculomegaly, Fetal[Text Word])) OR (Fetal Cerebral Ventriculomegalies[Text Word])) OR (Ventriculomegalies, Fetal Cerebral[Text Word])) OR (Ventriculomegaly, Fetal Cerebral[Text Word])) AND (((((((((Intraventricular hemorrhage[MeSH Terms]) OR (Cerebral Intraventricular Hemorrhages[Text Word])) OR (Hemorrhage, Cerebral Intraventricular[Text Word])) OR (Intraventricular Hemorrhage, Cerebral[Text Word])) OR (Cerebral Intraventricular Haemorrhage[Text Word])) OR (Cerebral Intraventricular Haemorrhages[Text Word])) OR (Haemorrhage, Cerebral Intraventricular[Text Word])) OR (Intraventricular Haemorrhage, Cerebral[Text Word])) OR (Intraventricular Haemorrhages, Cerebral[Text Word])).

**Search strategy in Cochrane Library:**

ID Search

#1 MeSH descriptor: [Hydrocephalus] explode all trees

#2 (hydrocephalus)

#3 (hydrocephaly)

#4 (cerebral ventriculomegaly)

#5 (cerebral ventriculomegalies)

#6 (ventriculomegalies, cerebral)

#7 (ventriculomegaly, cerebral)

#8 (communicating hydrocephalus)

#9 (hydrocephalus, communicating)

#10 (congenital hydrocephalus)

#11 (hydrocephalus, congenital)

#12 (hydrocephalus ex-vacuo)

#13 (hydrocephalus ex vacuo)

#14 (hydrocephalus ex-vacuos)

#15 (obstructive hydrocephalus)

#16 (hydrocephalus, obstructive)

#17 (post-traumatic hydrocephalus)

#18 (hydrocephalus, post-traumatic)

#19 (post traumatic hydrocephalus)

#20 (aqueductal stenosis)

#21 (aqueductal stenoses)

#22 (stenoses, aqueducta)

#23 (stenosis, aqueductal)

#24 (fetal cerebral ventriculomegaly)

#25 (cerebral ventriculomegalies, fetal)

#26 (cerebral ventriculomegaly, fetal)

#27 (fetal cerebral ventriculomegalies)

#28 (ventriculomegalies, fetal cerebral)

#29 (ventriculomegaly, fetal cerebral)

#30 #1 OR #2 OR #3 OR #4 OR #5 OR #6 OR #7 OR #8 OR #9 OR #10 OR #11 OR #12 OR #13 OR #14 OR #15 OR #16 OR #17 OR #18 OR #19 OR #20 OR #21 OR #22 OR #23 OR #24 OR #25 OR #26 OR #27 OR #28 OR #29

#31 MeSH descriptor: [Cerebral Intraventricular Hemorrhage] explode all trees

#32 (intraventricular hemorrhage)

#33 (cerebral intraventricular hemorrhages)

#34 (hemorrhage, cerebral intraventricular)

#35 (intraventricular hemorrhage, cerebral)

#36 (cerebral intraventricular haemorrhage)

#37 (cerebral intraventricular haemorrhages)

#38 (haemorrhage, cerebral intraventricular)

#39 (intraventricular haemorrhage, cerebral)

#40 (intraventricular haemorrhages, cerebral)

#41 #31 OR #32 OR #33 OR #34 OR #35 OR #36 OR #37 OR #38 OR #39 OR #40

#42 #30 AND #41

**Search Strategy in Web of Science:** (((((((((((((((((((((((((((((((TS= Hydrocephalus) OR (AB= Hydrocephaly)) OR (AB= Cerebral Ventriculomegaly)) OR (AB= Cerebral Ventriculomegalies)) OR (AB= Ventriculomegalies, Cerebral)) OR (AB= Ventriculomegaly, Cerebral)) OR (AB= Communicating Hydrocephalus)) OR (AB= Hydrocephalus, Communicating)) OR (AB= Congenital Hydrocephalus)) OR (AB= Hydrocephalus, Congenital)) OR (AB= Hydrocephalus Ex-Vacuo)) OR (AB= Hydrocephalus Ex Vacuo)) OR (AB= Hydrocephalus Ex-Vacuos)) OR (AB= Obstructive Hydrocephalus)) OR (AB= Hydrocephalus, Obstructive)) OR (AB= Post-Traumatic Hydrocephalus)) OR (AB= Hydrocephalus, Post-Traumatic)) OR (AB= Post Traumatic Hydrocephalus)) OR (AB= Aqueductal Stenosis)) OR (AB= Aqueductal Stenoses)) OR (AB= Stenoses, Aqueducta)) OR (AB= Stenosis, Aqueductal)) OR (AB= Fetal Cerebral Ventriculomegaly)) OR (AB=Cerebral Ventriculomegalies, Fetal)) OR (AB= Cerebral Ventriculomegaly, Fetal)) OR (AB= Fetal Cerebral Ventriculomegalies)) OR (AB= Ventriculomegalies, Fetal Cerebral)) OR (AB= Ventriculomegaly, Fetal Cerebral)) AND (((((((((TS=Intraventricular hemorrhage) OR (AB= Cerebral Intraventricular Hemorrhages)) OR (AB= Hemorrhage, Cerebral Intraventricular)) OR (AB= Intraventricular Hemorrhage, Cerebral)) OR (AB=Cerebral Intraventricular Haemorrhage)) OR (AB= Cerebral Intraventricular Haemorrhages)) OR (AB= Haemorrhage, Cerebral Intraventricular)) OR (AB= Intraventricular Haemorrhage, Cerebral)) OR AB= Intraventricular Haemorrhages, Cerebral))

**Appendix S2.** Detailed reasons for the exluded studies.

Review (n = 1):

Huttner HB, Nagel S, Tognoni E, Köhrmann M, Jüttler E, Orakcioglu B, Schellinger PD, Schwab S, Bardutzky J. Intracerebral hemorrhage with severe ventricular involvement: lumbar drainage for communicating hydrocephalus. Stroke. 2007 Jan;38(1):183-7. doi: 10.1161/01.STR.0000251795.02560.62. Epub 2006 Nov 22. PMID: 17122427.

Case reports or series (n = 6):

1. Sato M, Nakano M, Sasanuma J, Asari J, Watanabe K. An unusual hemorrhagic complication after ventriculostomy in a patient with lacunar infarcts. J Stroke Cerebrovasc Dis. 2004 Nov-Dec;13(6):280-2. doi: 10.1016/j.jstrokecerebrovasdis.2004.07.006. PMID: 17903988.
2. Huttner HB, Schwab S, Bardutzky J. Lumbar drainage for communicating hydrocephalus after ICH with ventricular hemorrhage. Neurocrit Care. 2006;5(3):193-6. doi: 10.1385/NCC:5:3:193. PMID: 17290087.
3. Jergović I, Budinčević H, Planjar-Prvan M, Bielen I. Transient Obstructive Hydrocephalus in Patients with Intracerebral Hemorrhage: Report of Two Cases. Acta Clin Croat. 2016 Sep;55(3):497-500. doi: 10.20471/acc.2016.55.03.21. PMID: 29046016.
4. Sindelar BD, Patel V, Chowdhry S, Bailes JE. A Case Report in Hemorrhagic Stroke: A Complex Disease Process and Requirement for a Multimodal Treatment Approach. Cureus. 2018 Jul 13;10(7):e2976. doi: 10.7759/cureus.2976. PMID: 30225183; PMCID: PMC6138459.
5. Tan LA, Kasliwal MK, An HS, Byrne RW. Obstructive Hydrocephalus Due to Intraventricular Hemorrhage After Incidental Durotomy During Lumbar Spine Surgery. Spine (Phila Pa 1976). 2018 Mar 1;43(5):E316-E319. doi: 10.1097/BRS.0000000000001074. PMID: 26208226.
6. Páscoa Pinheiro J, Carneiro DR, Matos D, Pereira R. Primary intraventricular haemorrhage: the role of frontal minicraniotomy and external ventricular drainage. BMJ Case Rep. 2021 Feb 9;14(2):e239448. doi: 10.1136/bcr-2020-239448. PMID: 33563669; PMCID: PMC7875280.

No valid outcomes (n = 5):

1. Siomin V, Weiner H, Wisoff J, Cinalli G, Pierre-Kahn A, Saint-Rose C, Abbott R, Elran H, Beni-Adani L, Ouaknine G, Constantini S. Repeat endoscopic third ventriculostomy: is it worth trying? Childs Nerv Syst. 2001 Sep;17(9):551-5. doi: 10.1007/s003810100475. PMID: 11585330.
2. Walcott BP, Berkhemer OA, Leslie-Mazwi TM, Chandra RV, Ogilvy CS, Yoo AJ. Multimodal endovascular treatment of a vertebrovertebral fistula presenting with subarachnoid hemorrhage and hydrocephalus. J Clin Neurosci. 2013 Sep;20(9):1295-8. doi: 10.1016/j.jocn.2013.01.006. Epub 2013 Jul 2. PMID: 23830589; PMCID: PMC3743960.
3. Andreasen TH, Holst AV, Lilja A, Andresen M, Bartek J Jr, Eskesen V, Juhler M. Valved or valveless ventriculoperitoneal shunting in the treatment of post-haemorrhagic hydrocephalus: a population-based consecutive cohort study. Acta Neurochir (Wien). 2016 Feb;158(2):261-70; discussion 270. doi: 10.1007/s00701-015-2659-2. Epub 2015 Dec 14. PMID: 26668079.
4. Luong CQ, Nguyen AD, Nguyen CV, Mai TD, Nguyen TA, Do SN, Dao PV, Pham HTM, Pham DT, Ngo HM, Nguyen QH, Nguyen DT, Tran TH, Le KV, Do NT, Ngo ND, Nguyen VD, Ngo HD, Hoang HB, Vu HV, Vu LT, Ngo BT, Nguyen BX, Khuong DQ, Nguyen DT, Vuong TX, Be TH, Gaberel T, Nguyen LV. Effectiveness of Combined External Ventricular Drainage with Intraventricular Fibrinolysis for the Treatment of Intraventricular Haemorrhage with Acute Obstructive Hydrocephalus. Cerebrovasc Dis Extra. 2019;9(2):77-89. doi: 10.1159/000501530. Epub 2019 Aug 13. PMID: 31408859; PMCID: PMC6751468.
5. Sun T, You C, Ma L, Yuan Y, Yang J, Tian M, Zhou Y, Guan J. Comparison of ventriculoperitoneal shunt to lumboperitoneal shunt in the treatment of posthemorrhagic hydrocephalus: A prospective, monocentric, non-randomized controlled trial. Medicine (Baltimore). 2020 Jul 2;99(27):e20528. doi: 10.1097/MD.0000000000020528. PMID: 32629633; PMCID: PMC7337583.

Unable to extract adult patients (n = 3):

1. Aldana PR, Kestle JR, Brockmeyer DL, Walker ML. Results of endoscopic septal fenestration in the treatment of isolated ventricular hydrocephalus. Pediatr Neurosurg. 2003 Jun;38(6):286-94. doi: 10.1159/000070412. PMID: 12759507.
2. Oertel JM, Mondorf Y, Baldauf J, Schroeder HW, Gaab MR. Endoscopic third ventriculostomy for obstructive hydrocephalus due to intracranial hemorrhage with intraventricular extension. J Neurosurg. 2009 Dec;111(6):1119-26. doi: 10.3171/2009.4.JNS081149. PMID: 19425883.
3. Bayrakli F, Erkek E, Kurtuncu M, Ozgen S. Intraventricular hemorrhage as an unusual presenting form of Sneddon syndrome. World Neurosurg. 2010 Apr;73(4):411-3. doi: 10.1016/j.wneu.2010.01.010. PMID: 20849802.

Other etiology (n = 2):

1. Hirashima C, Ohkuchi A, Matsubara S, Furukawa M, Watanabe T, Suzuki M. Hydrocephalus after intraventricular hemorrhage in eclamptic woman with HELLP syndrome. Hypertens Pregnancy. 2006;25(3):255-7. doi: 10.1080/10641950600913040. PMID: 17065045.
2. Komatsu F, Komatsu M, Wakuta N, Oshiro S, Tsugu H, Iwaasa M, Inoue T. Comparison of clinical outcomes of intraventricular hematoma between neuroendoscopic removal and extraventricular drainage. Neurol Med Chir (Tokyo). 2010;50(11):972-6. doi: 10.2176/nmc.50.972. PMID: 21123979.
